# Proprioception and neuromuscular control at return to sport after ankle surgery with the modified Broström procedure

**DOI:** 10.1038/s41598-021-04567-z

**Published:** 2022-01-12

**Authors:** Jin Hyuck Lee, Hae Woon Jung, Woo Young Jang

**Affiliations:** 1 Department of Sports Medical Center, Korea University College of Medicine, Anam Hospital, Seoul, Republic of Korea; 2 Department of Orthopedic Surgery, College of Medicine, Korea University, 73, Inchon-ro, Seongbuk-gu, Seoul, 02841 Republic of Korea; 3 Department of Pediatrics, Kyung Hee University Medical Center, Seoul, Republic of Korea

**Keywords:** Health care, Medical research

## Abstract

The modified Broström procedure (MBP) is an initial treatment for symptomatic chronic ankle instability (CAI) patients. This study aimed to compare the proprioception and neuromuscular control ability of both affected and unaffected ankles at the time of return to sports after MBP for patients with scores of normal controls. 75 individuals (40 who underwent MBP, 35 normal controls) participated. The dynamic balance test scores were significantly higher in the affected ankle of the patients than in the controls (1.5 ± 0.6° vs. 1.1 ± 0.4°, p < 0.003). The time to peak torque for dorsiflexion (60.8 ± 13.9 ms vs. 52.2 ± 17.5 ms, p < 0.022) and eversion (68.9 ± 19.1 ms vs. 59.3 ± 21.1 ms, p < 0.043) was significantly delayed in the affected ankle of the patients than in the controls. The dynamic balance test and time to peak torque in CAI patients remained significantly reduced at the time of return-to-sport after MBP. Clinicians and therapists should be aware of potential deficits in proprioception and neuromuscular control when determining the timing of return to sports after MBP.

## Introduction

The potential implications of chronic ankle instability (CAI) include mechanical joint instability, peroneal muscle weakness, and a lack of proprioception and neuromuscular control^1–5^. In particular, lateral ankle ligament injuries, including those of the anterior talofibular ligament (ATFL) and calcaneofibular ligament (CFL), can cause mechanical ankle instability (MAI), whereas proprioception and neuromuscular deficits can lead to functional ankle instability (FAI). Thus, MAI and FAI are a component of CAI^6,7^. Most patients with CAI can recover to their pre-injury activity levels with conservative treatment, but some clinicians argue that surgical intervention is required in 10–30% of patients in whom conservative treatment is unsuccessful^2,8,9^. For these reasons, the modified Broström procedure (MBP) is typically the initial treatment in symptomatic CAI patients in whom recovery was not attained with conservative treatment^10^.

Several studies have noted that postural control^11–14^ and peroneal muscle reaction time^11–13,15^ may be significant factors in assessing the recovery of proprioception and neuromuscular control in CAI patients after conservative or surgical treatment. While some authors have reported good clinical outcomes in most CAI patients after MBP^16^, patients can still experience subjective instability and persistent pain^17–19^, which may be due to proprioception and neuromuscular control deficits^5^. However, the extent of the recovery of proprioception and neuromuscular control after MBP for CAI has rarely been investigated. Furthermore, although the recommended timeframe for a return to non-contact sports after ligament surgery has generally been 3 months postoperative^20–23^, no studies have investigated the proprioception and neuromuscular control at 3 months after MBP. Therefore, this study aimed to compare the proprioception and neuromuscular control of both affected and unaffected ankles in CAI patients at 3 months after a MBP with scores for normal controls. We hypothesized that patients’ proprioception and neuromuscular control scores at 3 months after MBP would recover to the same level as that of normal controls.

## Methods

### Participant enrollment

**Figure 1:**
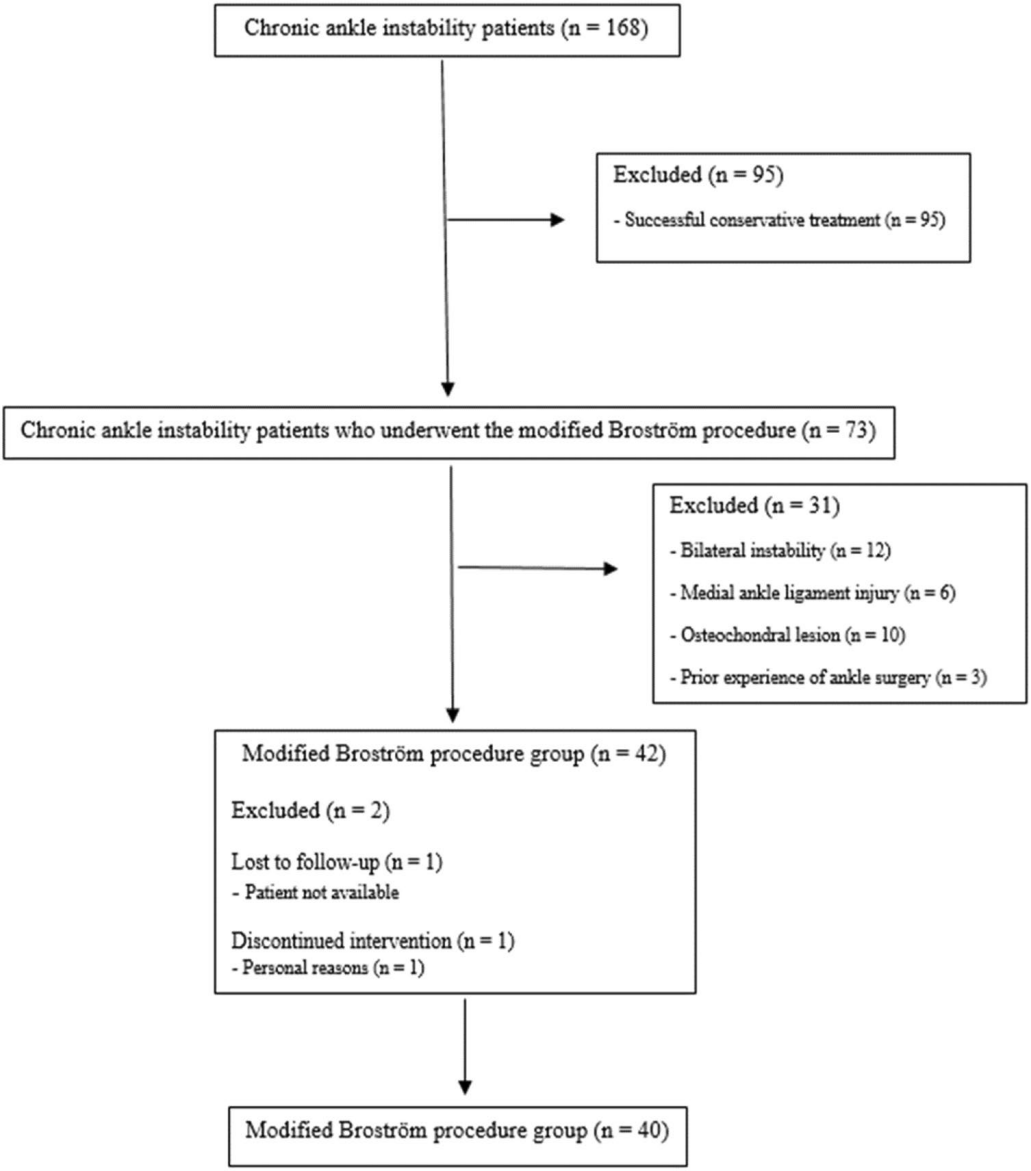
Flowchart of chronic ankle instability patients who underwent the modified Broström procedure.

Ethics approval was provided by the Institutional Review Board of the Korea University Anam Hospital (no: ED17143), and all research was performed in accordance with the relevant guidelines and regulations. Informed consent was obtained from all participants. A total of 73 patients who underwent MBP surgery between 2013 and 2017 among 168 patients with CAI were included in this retrospective case–control study. All patients underwent plain radiographic testing of both ankles to identify the lateral ankle ligament injuries. MAI was assessed as the presence of a talar tilt of more than 9° or an inter-ankle difference of more than 3° or 3 mm in the anterior drawer^24,25^. Any disagreements regarding physical examination or imaging findings were resolved by consensus of two experienced surgeons. All patients with CAI underwent conservative treatment for 3 months. If they reported constant symptomatic ankle instability and persistent pain despite conservative treatment, MBP was recommended for MAI with FAI. All patients who underwent MBP exhibited an ATFL tear with or without a CFL tear (grade 2 or 3 lesion) on magnetic resonance images and instability on stress plain radiographs^25^. Thirty-three patients were excluded for the following reasons (Fig. 1): bilateral ankle injury, medial ankle ligament injury, revised ankle surgery, refusal to be examined due to persistent postoperative pain, neuromuscular disease, acute ankle sprain, and osteochondral lesions. Then, 40 patients were allocated to the MBP group (12.4 weeks after MBP on average), and 35 normal control subjects were selected from our database of volunteers with no history of ankle joint injury. Finally, 75 participants (40 who underwent MBP vs. 35 normal controls) were enrolled.

### Evaluation tests

Recent studies reported that patients who underwent MBP returned to sports at 12 weeks^23,26^ and that proprioception and neuromuscular control are critical factors for a successful return to sports^12,27^. However, the terminology for proprioception and neuromuscular control in these studies has often been used interchangeably^12,27^. In the present study, proprioception and neuromuscular control tests were separately evaluated using postural stability and time to peak torque tests, respectively.

Since proprioception plays an important role in balance control^28^, the postural stability test was performed using the Biodex Stability System (BSS; Biodex Medical Systems, Shirley, NY, USA). While the static balance test involves maintaining the patient’s posture on a platform at level 12 (most stable), the dynamic balance test involves maintaining posture as the platform stability gradually decreases from level 12 (most stable) to level 1 (most unstable) with the level automatically declining every 1.66 s. The BSS provides a 20° platform tilt and 360° platform rotation. For this test, all participants stood barefoot with one leg on the platform while holding the opposite leg in a flexed position off the platform with the hands held behind the pelvis. Each test was performed for 2 trials, with rest time of 10 s between testing each leg. If the participant was unable to maintain a stable posture, that test was canceled. Each test was performed for 20 s to record postural stability parameters including the overall stability index (OSI, in degrees), with a lower OSI indicating better postural stability^24,29^.

In this study, to quantify the test–retest reliability for postural stability, intraclass correlation coefficients (ICCs) were calculated for two trials of static and dynamic balance. ICCs for static and dynamic balance were 0.90 and 0.85, respectively.

The neuromuscular control test was performed using a quantified isokinetic device (Biodex Multi-Joint System 4, Biodex Medical Systems Inc., Shirley, NY, USA). Neuromuscular control can be defined as the coordination or co-contraction of muscles for joint stability^30^. Neuromuscular control was measured using the time to peak torque, which reflects muscular reaction time and was defined as the arrival time (in milliseconds) from the initial contraction to the peak torque during muscle contraction^31^. Time to peak torque was recorded during 15 muscle contractions at 120°/s, with rest times of 30 s between feet and 1 min between tests performed in eversion and dorsiflexion.

### Postoperative rehabilitation protocol

Postoperative rehabilitation is divided into three phases at our clinic. All patients underwent cast immobilization for 2 weeks after the MBP. The initial phase started at 2–4 weeks postoperative and included range of motion and isometric muscle strengthening exercises with gradual full weight bearing. Starting at 6 weeks postoperative, the second phase introduced concentric and eccentric muscle strengthening of the hip, knee, and ankle joints and balance exercises to improve proprioception. Starting at 10 weeks postoperative, the third phase involved incremental muscle strengthening and plyometric exercises, including dynamic balance exercises, to improve neuromuscular control. Running and return to non-contact sports were allowed at 12 weeks. All participants performed the rehabilitation protocol for both ankles once or twice per week for 12 weeks postoperative and were taught the home rehabilitation program.

### Statistical analysis

Based on a previous study of postural stability in patients with ankle instability^24,32^, an intergroup OSI difference > 0.5° was considered a clinical difference. A power analysis was performed to determine the sample size, with an alpha level of 0.05 and a power of 0.8. The results of a pilot study with five ankles in each group indicated that 54 ankles were required to detect significant intergroup differences in OSI (> 0.5°). The power available to detect such differences in OSI was 0.810.

All continuous variables are described as mean ± SD. The Student’s *t*-test was used to compare proprioception (postural stability) and neuromuscular control (time to peak torque) the affected and unaffected ankles of the MBP versus normal control groups. A paired t-test was used to compare all pre- versus postoperative variables in the MBP group. The Shapiro test was used to determine whether continuous variables were normally distributed. The statistical significance was set at p < 0.05. The statistical analysis was performed using SPSS Statistics software (ver. 21.0; IBM, Chicago, IL, USA).

### Ethics approval and consent to participate

The study protocol was approved by Korea University Anam Hospital Institutional Review Board (No: ED 17143). All study participants provided written informed consent before being enrolled in this study.

## Results

Table 1 presents the participants’ demographic data. There were no significant differences in age, height, and weight between the 40 patients who underwent MBP and the 35 normal controls.

**Table 1:** Demographic data of subjects in the modified Broström procedure and normal control groups.

	Modified Broström procedure group (n = 40)	Normal control group (n = 35)	*p* value
Sex (male/female)	25/15	21/14	
Age (years)^a^	27.3 ± 3.6	24.8 ± 2.2	0.542
Height (cm)^a^	176.1 ± 0.8	175.3 ± 0.4	0.794
Weight (kg)^a^	61.3 ± 4.4	63.2 ± 6.5	0.626
Body mass index (kg/m^2^)^a^	22.1 ± 2.4	23.2 ± 3.6	0.512
Injured side (right/left)	28/12	21/14	
Sports and activity level, n (low:high)^a^	15:25	12:23	0.412

^a^Values expressed as mean ± standard deviation.

### Pre- versus postoperative outcome measures in the MBP group

**Figure 2:**
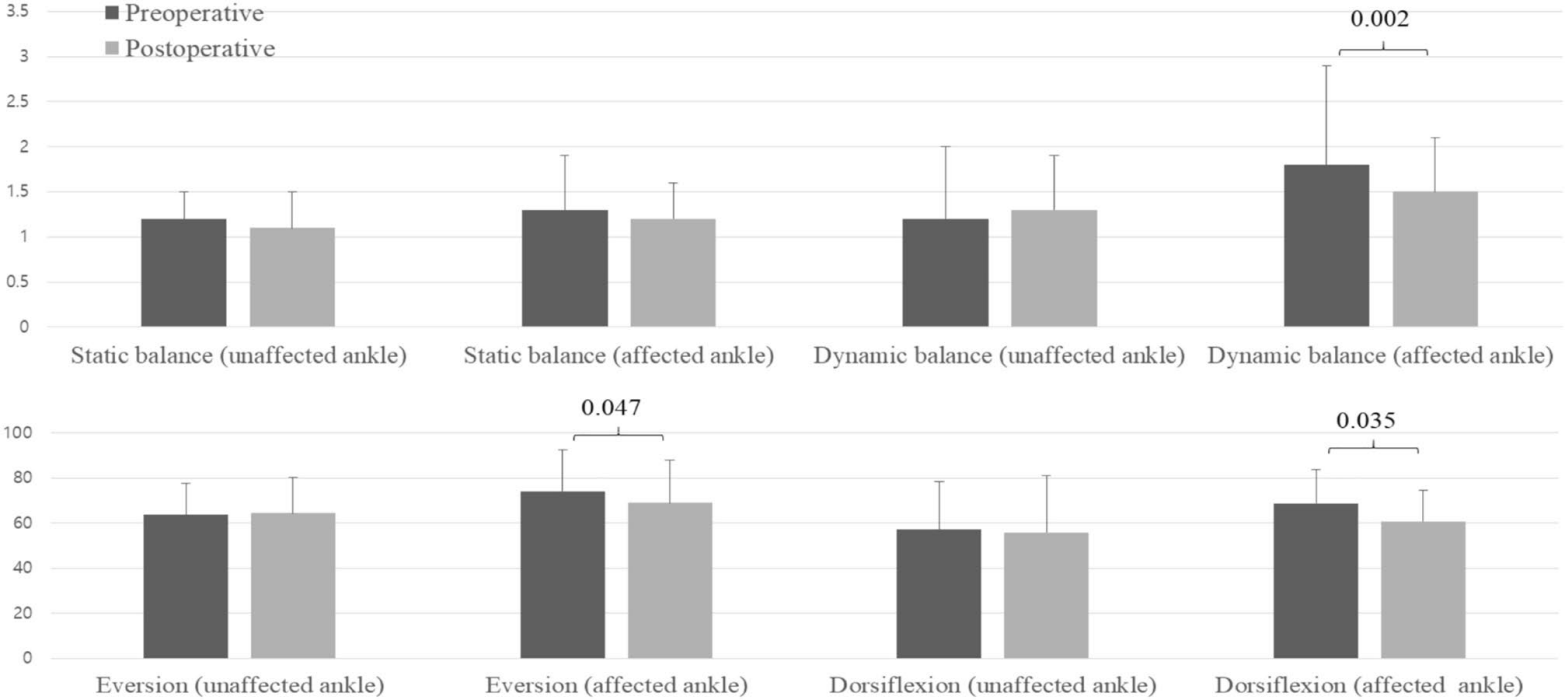
Pre- and postoperative postural stability (degrees) and time to peak torque (milliseconds) of the affected versus unaffected ankles between preoperative and postoperative in the MBP group. *MBP* modified Broström procedure.

Preoperative dynamic balance test scores of the affected ankles were significantly improved after MBP (1.8 ± 1.1° vs. 1.5 ± 0.6°, p = 0.002; Fig. 2), whereas the static balance test scores did not improve (p > 0.05). Preoperative time to peak torque for dorsiflexion and eversion of the affected ankles were significantly improved after MBP (dorsiflexion: 68.8 ± 15.0 ms vs. 60.8 ± 13.9 ms, p = 0.035; eversion: 76.1 ± 18.4 ms vs. 68.9 ± 19.1 ms, p = 0.047; Fig. 2), whereas those for the unaffected ankles remained unchanged (p > 0.05).

### Intergroup comparison of postoperative outcome measures

The static balance test showed no significant intergroup difference in the affected or unaffected ankles (p > 0.05; Table 2). The dynamic balance test scores were significantly higher for the affected ankles of the MBP group than for the ankles of the control group (1.5 ± 0.6° vs. 1.1 ± 0.4°; 95% confidence interval [CI]: 0.1–0.6; effect size: 0.784; p < 0.003; Table 2), with no difference noted in the unaffected ankles (p > 0.05). Times to peak torque for dorsiflexion and eversion were significantly delayed in the affected ankles of the MBP group versus those of the control group (dorsiflexion: 60.8 ± 13.9 ms vs. 52.2 ± 17.5 ms; 95% CI − 12.8 to 15.8; effect size: 0.544; p < 0.022; eversion: 68.9 ± 19.1 ms vs. 59.3 ± 21.1 ms; 95% CI 3.3–18.8; effect size: 0.477; p < 0.043; Table 3), and no intergroup differences were noted in the unaffected ankles (p > 0.05).

**Table 2:** Postural stability between the modified Broström procedure and normal control groups.

	Unaffected ankle			Affected ankle		
	Modified Broström procedure group	Normal control group	*p* value	Modified Broström procedure group	Normal control group	*p* value
Static balance, mean ± SD	1.1 ± 0.4	1.0 ± 0.4	0.806	1.2 ± 0.4	1.1 ± 0.4	0.245
MD, (95% CI)	0.1 (− 0.2, 0.2)			0.1 (− 0.1, 0.3)		
Effect size	0.250			0.249		
Dynamic balance, mean ± SD	1.3 ± 0.6	1.1 ± 0.3	0.088	1.5 ± 0.6	1.1 ± 0.4	**0.003**^**a**^
MD, 95% CI	0.2 (0, 0.4)			0.4 (0.1, 0.6)		
Effect size	0.421			0.784		

Significant values are in bold.

Values expressed as mean ± standard deviation.

The measurement units for the balance test was the degree.

*SD* Standard deviation, *MD* mean difference, *CI* confidence interval.

^a^Statistically significant.

**Table 3:** Time to peak torque between the modified Broström procedure and normal control groups.

	Unaffected ankle				Affected ankle		
	Modified Broström procedure group	Normal control group		*p* value	Modified Broström procedure group	Normal control group	*p* value
Dorsiflexion time to peak torque, mean ± SD	55.8 ± 25.1		56.4 ± 15.5	0.892	60.8 ± 13.9	52.2 ± 17.5	**0.022**^**a**^
MD, 95% CI	− 0.6 (− 10.4, 9.1)				8.6 (− 12.8, 15.8)		
Effect size	− 0.028				0.544		
Eversion time to peak torque, Mean ± SD	64.4 ± 15.8		56.0 ± 22.5	0.072	68.9 ± 19.1	59.3 ± 21.1	**0.043**^**a**^
MD, (95% CI)	8.4 (− 5.3, 17.2)				9.6 (3.3, 18.8)		
Effect size	0.432				0.477		

Significant values are in bold.

Values expressed as mean ± standard deviation.

The measurement units for time to peak torque tests was the millisecond.

*SD* Standard deviation, *MD* mean difference, *CI* confidence interval.

^a^Statistically significant.

## Discussion

The most important result of the present study was that dynamic balance test scores and times to peak torque of dorsiflexion and eversion were significantly reduced in the affected ankles of the MBP group versus those of the normal control group.

In the postural stability tests, dynamic balance test was significantly reduced in the affected ankles of the MBP group compared with those of the normal controls, except for static balance test. Although the reason for this result is unclear, it can likely be explained by mechanoreceptors, sensory receptors located in the ligaments that provide sensory information from external stimuli^33^. They include Pacinian corpuscles (rapidly adapting mechanoreceptors) and Ruffini endings, muscle spindles, and Golgi tendon organs (slowly adapting mechanoreceptors)^34,35^. Recent studies reported that, compared with Ruffini endings and Golgi tendon organs, Pacinian corpuscles predominate in the lateral ligaments of the human ankle^33,36^, which makes them especially capable of detecting motion and dynamic sense^37^. Therefore, rapidly adapting mechanoreceptors loss can affect dynamic postural stability on the dynamic balance test^33,36^, but not static postural stability. In addition, McKeon and Hertel, reported that the static balance test may not be an appropriate assessement to detect balance deficits in CAI patients^12^. Therefore, the dynamic balance test may be helpful in identifying balance deficits between CAI patients who underwent MBP and normal controls compared with the static balance test^38^. Another possible reason for this result may be insufficient ligament recovery. A previous study reported that lateral ankle ligament deficits may decrease postural stability due to increased mechanical instability^39^. In the present study, the dynamic postural stability test was performed 3 months after the MBP, and the improvement of mechanical stability seems to be insufficient in this time^40^.

Systematic review studies^41,42^ reported that a delayed peroneal reaction time may cause CAI. In the present study, times to peak torque as muscle reaction time for dorsiflexion and eversion were significantly increased in the affected ankles of the MBP group versus the ankles of the normal control groups. One possible explanation for the difference in results between the MBP and normal control groups is that the muscle reaction time may not be affected by surgical repair. As previously mentioned, rapidly adapting mechanoreceptors are prevalent (versus slowly adapting mechanoreceptors) in the lateral ankle ligament^33,36^. The MBP is performed to facilitate mechanical stability improvements, but muscle reaction time is detected by slowly adapting mechanoreceptors^43,44^. Therefore, the authors believe that the recovery of slowly adapting mechanoreceptors may be insufficient through surgical repair alone^45^. Furthermore, Li et al. reported that the muscle reaction times of dorsiflexion and eversion did not improve on electromyography (EMG) after MBP^46^, probably because the delayed muscle reaction time is not related to ligament injury or mechanical instability^47^. Given that the results of the present study were consistent with those of previous studies, we believe that proprioception and neuromuscular training should be considered postoperatively to improve muscular reaction time in dorsiflexion and eversion^45,48,49^.

This study had several limitations. First, it is possible that visual compensation may directly affect postural stability as reported by Li et al.^14^. However, in the present study, the control screen was covered to reduce any bias of the dynamic balance test. Second, we did not perform EMG in the neuromuscular response assessment. However, isokinetic devices are valid indirect measurement tools for the assessment of muscular response, including muscular reaction time, since they quantify the time to peak torque^31,50^. Finally, we did not evaluate clinical outcomes such as the Halasi score^51,52^ or the Foot and Ankle Ability Measure-sports score^53^; thus, further prospective studies including such clinical outcomes are necessary to confirm the optimal timing for a return to sports after MBP. Despite these limitations, to the best of our knowledge, this is the first study to determine the differences in proprioception and neuromuscular control between CAI patients who underwent MBP and normal controls.

In conclusion, dynamic balance test scores and times to peak torque of CAI patients remained significantly reduced at the timing of return-to-sport after MBP. Therefore, clinicians and therapists should be aware of potential proprioception and neuromuscular control deficits when determining the timing of return-to-sport after MBP.

## Data Availability

The datasets generated or analyzed during this study cannot be disclosed due to the standing policy of the Korea University Anam Hospital Research Ethics Board.
